# Speaking Concisely and Comprehensively: Importance of an Efficient Communication Model Between the Physician and the Patient

**DOI:** 10.7759/cureus.3156

**Published:** 2018-08-17

**Authors:** Abhishek Gupta

**Affiliations:** 1 Geriatrics, Center for Addiction and Mental Health/University of Toronto, Toronto, CAN

**Keywords:** patient-physician communication, nutritional counseling, language, efficiency

## Abstract

Ideal communication between a physician and a patient, where there are higher efficiency and the conveying of quality information, is the cornerstone of achieving meaningful health outcomes for the patient. Today, increasing constraints in the form of mandatory documentation, guideline-based requirements, and other bureaucratic necessities have limited the time that the physician can truly spend on communicating with the patient. As such, counseling often takes lower priority during a patient visit. To correct this deficiency, the modern physician must adapt to the new constraints to create a more-efficient communication model that incorporates greater literature and infographic-based technological usage with advanced preparation. It is a challenge that must be met in order to maintain a critical element of patient visits.

## Editorial

Traditional medical training has often focused on accurate and effective communication between physicians and patients. However, with documentation necessities, communiqués, lab reports, and other aspects of guideline-driven patient care have added nearly seven hours to a primary care clinician’s workday [1]. Routine, short primary care visits (15-20 minutes) are not only longer by one to two minutes but also involve increasingly complex medical issues as well as medication reviews. This is especially true for elderly patients, of whom 40% have three or more medications as well as three or more chronic medical conditions [1]. Given such an added workload over short durations, patient visits now have fewer healthcare issues addressed, a reduced patient understanding of pathology, excess emergency service usage, noncompliance with regimens, and overall decreased patient satisfaction. Further, physician burnout rates and medical errors are increasing concurrently [1]. As such, efficient communication now must also be part of physician training in order to maintain or improve patient healthcare outcomes.

The medical knowledge gap between the physician and the patient is a severe limiter on an effective physician-patient relationship. Adding in linguistic differences and time constraints on interviews, the difficulties are compounded further. In this area particularly, an efficient communication model can serve to improve the healthcare outcomes of even a short patient visit.

To portray this, I would like to share my experience when I was a sub-intern at a community clinic in Toronto attending a predominantly South Asian demographic. Being a South Asian immigrant and having a personal fluency of multiple dialects, I was often allowed to conduct patient interviews as an official interpreter. As multiple cases of Type 2 Diabetes Mellitus (DM2) and hypertension were often encountered, I found myself often explaining the need for medication and the positive effect of lifestyle modifications. Given temporal constraints, I was initially chided by my supervisors for lengthy patient education sessions that often resulted in patient visit backlogs. With time, however, I developed an efficient model that explained, using a patient’s own ethnic dialect and infographics, the most common medical diagnoses within five minutes. This model allowed the patient to more effectively anticipate the health-based consequences of their actions. For example, using animated videos of diabetic nephropathy’s pathophysiology allowed a patient to realistically imagine the consequences of persistent hypertension with DM2 and, therefore, improved compliance with their lisinopril drug use as well as low-salt diet regimen. As such, it resulted in a significant improvement in patient compliance with lifestyle modification and medication regimens. It is also important to note that I did have a significant competency in terms of the ethnic and cultural aspects of patient demographics. Such an aspect plays a key role in improving communication. 

An efficient model for patient education and counseling delivery, such as in the example above, requires the advanced preparation of medical literature in an easily understandable format. An ideal format should incorporate visual aids and local languages to reduce linguistic miscommunication risks. As appropriate counseling or screening during primary care visits account for 2.6 to 4.2 minutes, an efficient counseling session should work within such a time frame [2]. Additionally, common primary care issues, such as DM2, essential hypertension, and arthropathies, should especially be targeted and prepared for. Most importantly, it is the physician's prerogative to use his or her own experience in estimating how much medical information is ideal for a condition and is suitable for a patient's level of literacy and capacity. Such innovations are not necessarily technological in nature or even have any additional financial burden. For example, physicians who have Hispanic patients with limited English proficiency (LEP) can obtain pamphlets on low-sodium diets in Spanish from the Center of Disease Control website in advance of a patient visit. Similarly, an adolescent patient may favor animated videos on the physiological mechanism of immunity for better compliance with their annual flu vaccine regimen. Such tools require minimal technical knowledge and are often provided at no cost by governmental and non-governmental organizations. The key is to analyze what patients routinely request and need in terms of counseling, estimating their medical and English literacy, and preparing suitable educational materials in advance.

Physicians, especially those in training, can be at the forefront of this innovation. The new generation of medical residents and students should practice efficient and concise speech strategies when learning to speak with patients. Being more technologically informed, they have the additional advantage in terms of an awareness of multimedia educational resources on various Internet-based platforms. Practical medical training would certainly benefit from patient simulations that have realistic patient demands and time limitations.

In conclusion, an effective and comprehensive model of patient-physician interaction must incorporate efficiency in order to avoid compromising the counseling component. With new guideline requirements and temporal constraints, the traditional models of patient education must be reformed. Innovation and clinical experience are the keys to estimating the necessary patient counseling parameters and delivering the appropriate counseling. Incorporating new multimedia technologies, layman language, and a concise format on a multilingual platform would make an ideal, efficient communication model.
